# Vision Health-Related Quality of Life in Chinese Glaucoma Patients

**DOI:** 10.1155/2015/271425

**Published:** 2015-10-07

**Authors:** Lei Zuo, Haidong Zou, Jianhong Zhang, Xinfeng Fei, Xun Xu

**Affiliations:** ^1^Department of Ophthalmology, Shanghai General Hospital, Nanjing Medical University, Shanghai 200080, China; ^2^Department of Ophthalmology, Branch of Shanghai First People's Hospital, Shanghai 200081, China; ^3^Department of Ophthalmology, Shanghai General Hospital, Shanghai Jiao Tong University, Shanghai 200080, China

## Abstract

This cross-sectional study evaluated VRQOL in Chinese glaucoma patients and the potential factors influencing VRQOL. The VRQOL was assessed using the Chinese-version low vision quality of life questionnaire. Visual field loss was classified by the Hodapp-Parrish-Anderson method. The correlations of VRQOL to the best corrected visual acuity and the VF loss were investigated. The potential impact factors to VRQOL of glaucoma patients were screened by single factor analysis and were further analyzed by multiple regression analysis. There were significant differences in VRQOL scores between mild VF loss group and moderate VF loss group, moderate VF loss group and severe VF loss group, and mild VF loss group and severe VF loss group according to the better eye. In multiple linear regression, the binocular weighted average BCVA significantly affected the VRQOL scores. Binocular MD was the second influencing factor. In logistic regression, binocular severe VF loss and stroke were significantly associated with abnormal VRQOL. Education was the next influencing factor. This study showed that visual acuity correlated linearly with VRQOL, and VF loss might reach a certain level, correlating with abnormal VRQOL scores. Stroke was significantly associated with abnormal VRQOL.

## 1. Introduction

Glaucoma, a group of eye diseases that permanently damage visual function [1], can impact patient quality of life [2] adversely [3–5]. Ophthalmologists have been working on the best treatment for glaucoma patients and mitigating the adverse impact. Previous studies have investigated the life quality of glaucoma patients, suggesting a relationship between visual field defects and impaired quality of life in patients with glaucoma [6–8]. Furthermore, the association between rates of binocular visual field loss and vision-related quality of life in glaucoma was observed [9]; special attention was paid to the quality of life of young patients with glaucoma [10]; and Globe et al. noticed that self-reported systemic comorbid diseases were associated with self-reported visual function [11]. Understanding of influencing factor to quality of life of the patients will ultimately benefit glaucoma treatment.

Epidemiologic studies in China showed that the overall prevalence rate of primary glaucoma was 0.56% and that of populations over 50 years of age was 2.07% [12]. The blindness rate of glaucoma was 9.04–10% [13]. Lee et al. [14] investigated the association between clinical parameters and quality of life in Chinese primary open angle glaucoma patients (using the Glaucoma Quality of Life-15 Questionnaire (GQL-15)). Kong et al. [15] found that the level of understanding about glaucoma was associated with psychological disturbance and quality of life (using the 25-Item National Eye Institute Visual Function Questionnaire (NEI-VFQ 25) [16]).

In the present study, we assessed vision health-related quality of life (VRQOL) [17] in Chinese glaucoma patients using Chinese-version low vision quality of life questionnaire (CLVQOL) [18] and made comprehensive analysis on screening the potential influencing factors (such as visual field damage, glaucoma type (primary open angle or angle close), age, and self-reported comorbidities) to VRQOL. The CLVQOL questionnaire was originally acquired from the low vision health-related quality of life questionnaire (LVQOL) [17] and translated into a Chinese version that was modified and culturally adapted for the Chinese patients [18]. We hoped our results could provide reference for clinical better understanding of glaucoma patients and developing suitable therapeutic strategy for them.

## 2. Methods

### 2.1. Study Patients

There were 202 glaucoma patients who met eligibility criteria and agreed to participate in the study, and these patients were enrolled at the glaucoma clinic at the Branch of Shanghai First People's Hospital from January 1, 2013, through June 31, 2013. The investigation was approved by the hospital ethics committee. All methods adhered to the Declaration of Helsinki. All participants gave their written informed consent.

The inclusion criteria were adult patients (18 years old and above) with glaucoma diagnosis based on glaucomatous disc cupping and reproducible visual field damage detected by automated static perimetry (the Humphrey Visual Field Analyzer) in one or both eyes [4, 19]. There were 2 kinds of glaucoma: primary open angle glaucoma and primary angle closure glaucoma [19] in our study. The exclusion criteria were as follows: (1) secondary glaucoma; (2) any other coexisting ocular condition that could impair visual function (e.g., clinically significant cataract, macular degeneration, or any other ophthalmic condition); (3) incisional ocular surgery or laser treatment in past except antiglaucoma surgery and laser therapy; and (4) disability in a visual field test due to cognitive impairment.

At each follow-up visit, patients underwent a comprehensive ophthalmic examination, including review of medical history, best-corrected visual acuity, slit lamp biomicroscopy, intraocular pressure measurement using noncontact tonometry, gonioscopy, stereoscopic optic disc photography (Canon, CR-1 Mark II), visual fields test, and optic nerve head assessment in optical coherence tomography (Stratus OCT, Carl Zeiss Meditec, CA). The type of local ophthalmic medication was also noted.

### 2.2. Binocular Visual Fields

Visual fields (VF) test was performed using the Humphrey Visual Field Analyzer (Humphrey Instruments, Zeiss, CA). Humphrey central 30-2 threshold test plotted the central 30 degrees of visual field in both eyes. Only reliable tests (≤33% fixation losses and false-negative results and ≤15% false-positive results) were included. Visual fields were reviewed and excluded in the presence of artifacts such as eyelid or rim artifacts, fatigue effects, inattention, or inappropriate fixation. Visual fields were also reviewed for the presence of abnormalities that could indicate diseases other than glaucoma, such as homonymous hemianopia.

Mean deviation (MD) scores were used to assess the severity of VF loss. For the purpose of the statistical analysis, the binocular VF loss was classified into 3 groups according to MD: group 1 (mild: −6 dB < MD < −3 dB), group 2 (moderate: −12 dB < MD < −6 dB), or group 3 (severe: MD < −12 dB) (the Hodapp-Parrish-Anderson method [20]), which was used for the better eye and worse eye. According to spearman correlation and linear regression, monocular data of MD values was transformed to binocular data using a formula for binocular summation, as suggested by Nelson-Quigg et al. [21]:

(1) Binocular  Sensitivity=Sensitivity  R  eye2+Sensitivity  L  eye2.

### 2.3. Visual Function Questionnaire

The VRQOL was evaluated using the CLVQOL questionnaire [17, 18]. The scale consists of four scales: general vision and lighting (reading road signs or watching TV), mobility (outdoor activities and crossing a street with traffic), psychological adjustment (expectations on quality of life and perceived visual acuity), and reading and fine work and activities of daily living (reading the clock, reading one's own handwriting, and daily activities), including 25 items. Each item was scored using a numeric scale ranging from 0 (worst) to 5 (best). The total highest score was 125, and the higher the score, the better the quality of life [17, 18]. The questionnaires were completed through face-to-face patient interviews conducted by two well-trained investigators.

### 2.4. Demographic and Clinical Variables

Demographic and clinical questionnaires were also administered to patients concurrent with the CLVQOL questionnaire. These questionnaires contained a survey of demographics, history of glaucoma, marital status, residence, educational level, and history of topical antiglaucomatous treatment.

Self-reported systemic comorbidities were investigated as follows: hypertension, diabetes mellitus, stroke, and other systemic comorbidities such as asthma, cancers, and heart disease.

Visual acuity was measured using the Snellen visual acuity chart. The best corrected visual acuity (BCVA) (subjective optometry) was converted into the minimum angle of resolution (logMAR) vision. When Snellen visual acuity was less than 0.01, the visual acuity of “counting fingers” perception was defined as logMAR2.2, “hand-motion” as logMAR2.3, and “light perception” as logMAR2.5 [22]. Monocular data of logMAR BCVA was transformed to binocular weighted average BCVA with the weight of the better eye and the worse eye taken as 0.75 and 0.25, respectively (as recommended by Scott et al. [23]).

### 2.5. Statistical Analysis

Descriptive statistics included the mean and standard deviation (SD) for variables. The Spearman correlation was used to analyze the correlation between the binocular weighted average logMAR BCVA and the VRQOL scores and between the binocular MD and the VRQOL scores. When equal variances were assumed, the 1-way ANOVA or *χ* ^2^ test was used to process the impact of demographic and clinical variables on the VRQOL scores change. When equal variances were not assumed, a nonparametric test or Fisher's exact 2-tailed test was used.

After single factor analysis of demographic and clinical variables, the statistically significant or nearly significant results were screened out. Then multiple linear regression was used to analyze the impact of linear variables on the VRQOL score changes. Logistic regression was used to analyze dichotomous variables; VRQOL scores were considered to be the dependent variable. These variables were categorized into dichotomous variables as follows: marital status (married (yes/no)), residence (urban (yes/no)), education (more than secondary school degree (yes/no)), both eyes MD < −12 dB (yes/no), at least 3 years glaucoma history (yes/no), stroke (yes/no), diabetes mellitus (yes/no), and VRQOL (at least mean value (yes/no)).

The level of statistical significance was set at 0.05. All statistical analyses were performed using SPSS 11.0 (SPSS Inc., Chicago, IL, USA).

## 3. Results

### 3.1. Patient Demographic Characteristics and Clinical Variables with VRQOL

**Figure 1:**
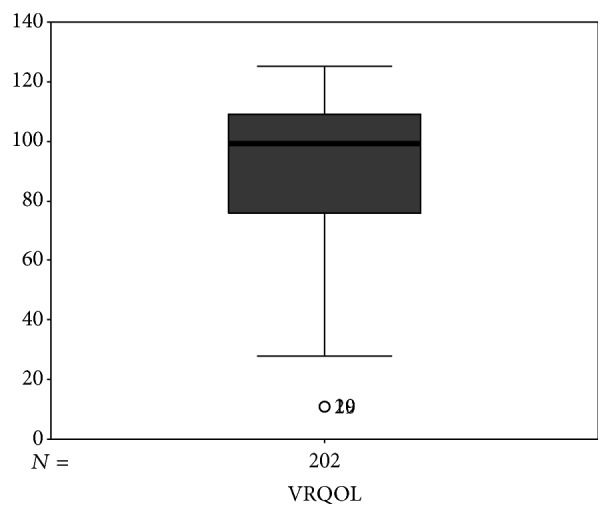
Boxplot showing the range of visual health-related quality of life (VRQOL).

There were 92 men and 110 women enrolled in this study, with a mean age (mean (SD)) of 69.49 (12.04) years, ranging from 31 to 89 years. The mean VRQOL score was 92.08 (23.97), ranging from 11 to 125 (Figure 1). VRQOL differences between 50–59 years and 60–69 years and between 50–59 years and 70–79 years were significant (*P* = 0.032, 0.018); differences in VRQOL between other age groups were not significant (all *P* > 0.10). When VRQOL of patients with different education levels (level 1 = illiterate and primary school, level 2 = secondary school, and level 3 = more than secondary school) were compared, there was a significant difference in VRQOL between level 1 and level 3 (*P* = 0.027); VRQOL differences between the other levels were not significant (all *P* > 0.10). When VRQOL of patients with different glaucoma durations (≤3 months, 3–12 months, 1–3 years, and >3 years) were compared, the difference was found to be significant between the VRQOL scores of patients who had been diagnosed with glaucoma within the past 3 months and those of patients with a 3-year course (*P* = 0.045); no remarkable differences were observed in VRQOL scores of other durations of glaucoma (all *P* > 0.10) (Table 1).

#### 3.1.1. Spearman Correlation

The Spearman correlation coefficient between the binocular weighted average logMAR BCVA and VRQOL scores was 0.572 (*P* < 0.001), and correlation coefficient between the binocular MD and VRQOL scores was −0.490 (*P* < 0.001) (Table 2).

*Binocular VF Loss and VRQOL*. When VRQOL results of patients in the 3 VF loss groups according to the better eye were compared, there were significant differences between group 1 and group 2, group 2 and group 3, and group 1 and group 3 (all *P* = 0.014, 0.016, <0.001). When the VRQOL of the 3 groups according to the worse eye were compared, there was no difference between group 1 and group 2 (*P* = 0.509), but there was a significant difference between group 2 and group 3 and group 1 and group 3 (*P* = 0.015, 0.001) (Table 3).

*Glaucoma Type and VRQOL*. When VRQOL, binocular weighted logMAR BCVA, and binocular MD of different glaucoma type groups were compared, there were no differences (all *P* > 0.10) (Table 4).

### 3.2. Analysis of Multiple Impact Factors after Screening

*Denied Factors*. These included gender, the type of glaucoma, the number of antiglaucoma instillations, and previous antiglaucoma surgery/laser. These factors were not analyzed further.

*Possible Impact Factors*. These included age, education, marital status, glaucoma duration, and systemic comorbidity (diabetes mellitus, stroke). These factors were analyzed further.

*Positive Factors*. These included binocular weighted average logMAR BCVA, binocular visual field loss, and residence. These factors were analyzed further.

In multiple linear regression, binocular weighted BCVA impacted VRQOL scores significantly (*P* < 0.001), and binocular MD was the next factor (*P* = 0.063).

In logistic regression, severe binocular VF loss (both eyes MD < −12 dB) and stroke were significantly associated with abnormal VRQOL (*P* = 0.004, 0.016). More than secondary school degree was the secondary factor (*P* = 0.052) (Table 5).

*Example of 2 Patients*

*Patient A*. Patient A was a married 72-year-old female, with primary angle closure glaucoma; her residence was urban area; her education level was secondary school; and her medical history was as follows: 10 years after bilateral trabeculectomy, not using any antiglaucoma eye drop; MD: right eye: −5.75 dB, left eye: −6.63 dB; logMAR BCVA: right eye: 0.00, left eye: 0.00; and VRQOL score: 107.

*Patient B*. Patient B was a married 65-year-old male, with primary open angle glaucoma; his residence was urban area; his education level was more than secondary school; and his medical history was as follows: glaucoma duration for 11 years; using ≥3 kinds of antiglaucoma eye drop; self-reported systemic comorbidity: diabetes mellitus; MD: right eye: −27.52 dB, left eye: −29.76 dB; logMAR BCVA: right eye: 0.92, left eye: 0.83; and VRQOL score: 59.

## 4. Discussion

This cross-sectional study evaluated VRQOL in Chinese glaucoma patients and the potential factors influencing VRQOL. Here, we observed the demographic characteristics and clinical data of 202 Chinese glaucoma patients and analyzed the correlation between these variables and VRQOL.

### 4.1. Relationship between VRQOL and Visual Health in Glaucoma Patients

Spearman correlation analysis showed that VRQOL and binocular weighted average BCVA were closely related. In multiple linear regression, binocular weighted BCVA had a significant effect on VRQOL. VA and VRQOL of glaucoma patients were shown to have a direct linear correlation [14].

When binocular MD was regarded as a linear variable, the effect on VRQOL showed a trend toward significance. However, when binocular VF loss (binocular MD < −12 dB (yes/no)) was regarded as a dichotomous variable using logistic regression, the association with abnormal VRQOL was significant. This showed that binocular VF loss should reach a certain level, which could greatly affect the VRQOL of glaucoma patients [4–7].

After VF loss was further divided into groups according to VF loss stage [20, 24, 25], a significant relationship between VRQOL and VF loss stage was observed using the four subscales and in the better eye [26]. There was a significant influence on “mobility” subscale and “reading, fine work, and activities of daily living” subscale and total VRQOL scores with mild VF loss in the better eye that changed to moderate VF loss. However, there was a significant influence on the same subscales and total VRQOL scores with moderate VF loss of the worse eye that changed to severe VF loss. These results suggested that the better eye was more sensitive to visual field damage than the worse eye. As these results are shown, it is reasonable to believe that glaucoma patients sometimes neglect early VF damage.

In our study, the high correlation of the CDR with VRQOL was not found (better eye: 0.281, worse eye: 0.313) Our research suggested that the CDR had no significant relationship with patient quality of life [9, 27], and patients paid more attention on visual acuity and visual field results rather than OCT, though the defects of retinal nerve fiber layer were the important signal to be concerned by eye doctors.

### 4.2. The Effects of Glaucoma Patient Demographic Characteristics on VRQOL

The current research found that the relationship between age and VRQOL in glaucoma patients was more complicated than previous studies [6, 9]. The relationship might be influenced by the patients' psychological and environmental factors. Young people face pressures from life, study, and work, and if there was visual function damage or the threat of damage, the psychological impact would be great (Gupta et al. [28] used the Time-Tradeoff method to observe utility values among glaucoma patients and found that juveniles were willing to give up more years to spend the rest of their living years with perfect vision and free of glaucoma, compared with adult patients). However, elderly people, especially after retirement, showed a partial reduction in the pressures mentioned above. This reduction in pressures would be beneficial for the VRQOL. The coincidence degree of decline in VRQOL of glaucoma patients with age growth might be worse than other age related eye diseases [29, 30].

Labiris et al. [27] indicated that a higher educational background was positively correlated with higher vision-specific QOL scores, but Lisboa et al. [9] found that an educational level of at least high school completion had no significant effect on vision-related QOL. In a single analysis of the present study, the VRQOL score of glaucoma patients with an educational level of more than a secondary school degree was higher than that of the illiterate or primary school educational level of patients. In multiple linear regression, there was no linear relationship between education and VRQOL. However, using logistic regression, when education was regarded as dichotomous variable and was analyzed together with other variables, we found that more than a secondary school degree educational level was a nearly significant impact factor on abnormal VRQOL in glaucoma patients. Therefore, education that reaches a certain level could somewhat improve the VRQOL of glaucoma patients.

There was a significant effect of residence on VRQOL of glaucoma patients, and the effect of marital status showed a trend toward significance in single analysis of our study. It seemed that VRQOL of glaucoma patients living in urban areas was better than that of patients living in rural areas, and VRQOL of married glaucoma patients was probably better than that of patients who were unmarried. Multivariable regression showed that when these 2 factors were analyzed together with items such as education, visual field loss, and systemic comorbidity, there were no significant effects. Thus, residence and marital status might not be as important as other factors when they were considered together [9, 27].

### 4.3. The Influence of Medical Conditions and Other Factors on VRQOL of Glaucoma Patients

Our cross-sectional study showed that the change in the VRQOL value with the change in glaucoma duration was not statistically significant. Glaucoma patients' VRQOL was closely related to vision activity and visual field damage, and VRQOL and duration of the disease had no direct relationship.

This study indicated that there were no differences between the VRQOL of primary angle close glaucoma and primary open angle glaucoma [8]. Our results showed that the patients are not interested in whether their glaucoma type is the open or closed angle type, but rather their concern is the impact of the disease on their quality of life.

In this study, previous glaucoma surgery/laser had no impact on the VRQOL scores of glaucoma patients. The number of antiglaucoma eye drops also had no impact. This result was similar to that of previous reports, whose aim did not involve evaluating the side effects of glaucoma treatments [14, 27].

This study showed that whether VRQOL scores reached mean value was significantly influenced by stroke. In the Los Angeles Latino Eye Study, systemic comorbidity weighted index of glaucoma patients was analyzed. They indicated that the weighted index of stroke or brain hemorrhage was 2.06, that of diabetes mellitus was 1.80, and that of high blood pressure was 1.06. Stroke or brain hemorrhage had the greatest effect on glaucoma patients' VRQOL compared with other self-reported systemic comorbidities [11]. Our study similarly found that stroke could significantly affect the VRQOL of glaucoma patients. An explanation for this might be that stroke was significantly associated with visual impairment and low physical function [11]. Additionally, glaucoma patients in our study with high blood pressure had relatively high VRQOL, refracting their insufficient attention to hypertension.

## 5. Limitations

Our study has limitations. First, sample size was relatively small, and all patients were recruited from a single eye institute; this might cause selection bias. Second, our study was cross-sectional study; however, information obtained from longitudinal observation is likely to reduce the interindividual variability and possible effects of compensatory mechanisms and provides more robust evaluation on the association between variables and VRQOL [9, 31, 32]. Third, if more than one questionnaire (e.g., the Short-Form 36 Health Survey (SF-36) [33], which is used to assess the general health status of glaucoma patients [34], and the Glaucoma Symptom Scale (GSS) [35], a measure to assess the symptoms associated with glaucoma and its management [36]) is used simultaneously for the survey, the effects of glaucoma on the patient could be understood on different levels [37], and further studies could be performed to observe the difference [38] between CLVQOL and NEI VFQ-25 [16] or between CLVQOL and the Visual Activities Questionnaire (VAQ) [39], exploring which questionnaire can more exactly describe VRQOL for Chinese glaucoma patients.

In the analysis of the survey results, we should also note that normal variations in personality characteristics will influence how patients report their VRQOL [40].

Media opacity such as cataract can influence VRQOL; patients with obvious cataract were excluded from the study, but we did not use any classification (e.g., Lens Opacity Classification System III (LOCS III) [41]). It was limitation in the study.

In summary, the clinical eye doctors could treat, guide, and help glaucoma patients, manage therapeutic strategy to preserve or improve visual ability, and prevent visual field impairment. Ophthalmologists should also keep their patients well informed of the necessary knowledge about glaucoma [42], reduce their risk of stroke, and thereby protect their VRQOL.

## Figures and Tables

**Table 1:** Patient's demographic characteristics and clinical variables.

Variable	Number of patients	VRQOL score mean (SD)	*P* values
All	202	92.08 (23.97)	
Binocular weighted average BCVA	0.48 (0.52), 0.00 to 2.35		
BCVA for better eye	0.37 (0.49), 0.00 to 2.30		<0.001
<0.3 (>20/40)	122	102.80 (15.87)	
0.3 to 1.0 (20/200 to 20/40)	62	86.90 (20.42)	
>1.0 (<20/200)	18	44.00 (17.41)	
BCVA for worse eye	0.81 (0.81), 0.00 to 2.50		<0.001
<0.3 (>20/40)	60	105.53 (14.0)	
0.3 to 1.0 (20/200 to 20/40)	88	94.16 (17.94)	
>1.0 (<20/200)	54	73.74 (29.84)	
Binocular MD (dB value)	18.14 (12.09), 3.82 to 45.28		
MD for better eye (dB)	−8.76 (8.23), −31.00 to 1.37		<0.001
>−6	106	101.94 (16.25)	
−6 to −12	46	91.35 (19.19)	
<−12	50	71.84 (29.03)	
MD for worse eye (dB)	−15.28 (9.88), −33.19 to −2.98		0.001
>−6	48	104.17 (13.57)	
−6 to −12	44	100.18 (18.87)	
<−12	110	83.5 (26.10)	
Age (year)	69.49 (12.04), 31 to 89		0.151
≤49	14	98.29 (19.59)	
50–59	26	106.69 (15.13)	
60–69	52	89.23 (24.20)	
70–79	60	87.77 (23.34)	
≥80	50	90.88 (27.48)	
Gender			0.812
Male	92	91.43 (28.08)	
Female	110	92.62 (20.15)	
Education			0.079
Illiterate and primary school	36	81.67 (26.05)	
Secondary school	50	90.52 (23.01)	
Secondary school+	116	95.98 (23.06)	
Residence			0.002
Rural area	12	56.83 (27.72)	
Urban area	190	94.31 (22.05)	
Marital status (married)			0.085
Yes	152	95.07 (21.84)	
No	50	83.00 (28.94)	
Duration of glaucoma			0.222
≤3 months	20	105.00 (19.75)	
3–12 months	36	92.94 (21.50)	
1–3 years	44	94.32 (21.97)	
>3 years	102	88.27 (25.85)	
Previous antiglaucoma surgery/laser			0.867
Yes	42	93.90 (16.82)	
No	160	91.60 (25.58)	
Antiglaucoma eye drop (type)			0.869
0	56	91.57 (21.21)	
1	62	95.03 (20.57)	
2	68	90.29 (28.11)	
≥3	16	90.00 (29.52)	
Self-reported systemic comorbidity			0.072
No	70	93.00 (21.10)	
High blood pressure	56	99.71 (15.84)	
Diabetes mellitus	28	83.50 (24.41)	
Stroke	16	74.63 (25.73)	
Other systemic diseases	32	92.81 (34.68)	

VRQOL: visual health-related quality of life; SD: standard deviation; BCVA: best corrected visual acuity; MD: mean defect.

**Table 2:** Correlation between Chinese-version low vision quality of life (CLVQOL) questionnaire score and visual severity score.

	Spearman correlation	*P* value
BCVA		
Better eye	−0.614	<0.001
Worse eye	−0.483	<0.001
Binocular weighted average	−0.572	<0.001
MD		
Better eye	0.467	<0.001
Worse eye	0.491	<0.001
Binocular	−0.490	<0.001
PSD		
Better eye	−0.211	0.053
Worse eye	−0.078	0.487
VFI		
Better eye	0.447	<0.001
Worse eye	0.443	<0.001
CDR		
Better eye	−0.280	0.006
Worse eye	−0.313	0.003

VRQOL = vision health-related quality of life; BCVA = best corrected visual acuity; MD = mean defect; PSD = pattern standard deviation; VFI = visual field index; CDR = cup to disk ration.

**Table 3:** Relationship between vision health-related quality of life (VRQOL) score calculated for separate factors and visual fields (VF) loss.

	VRQOL score% (SD)	Mild VF loss	Moderate VF loss	Severe VF loss
		*P* value	*P* value	*P* value
Better eye				
General vision and lighting (GL)				
Mild VF loss	76.17 (15.57)	—	0.114	0.001^*∗*^
Moderate VF loss	68.94 (17.14)	0.114	—	0.096
Severe VF loss	56.11 (25.91)	0.001^*∗*^	0.096	—
Mobility (M)				
Mild VF loss	85.44 (15.16)	—	0.012^*∗*^	<0.001^*∗*^
Moderate VF loss	77.04 (15.52)	0.012^*∗*^	—	0.017^*∗*^
Severe VF loss	59.84 (24.72)	<0.001^*∗*^	0.017^*∗*^	—
Psychological adjustment (PA)				
Mild VF loss	79.35 (12.90)	—	0.066	0.001^*∗*^
Moderate VF loss	71.50 (17.75)	0.066	—	0.126
Severe VF loss	61.60 (23.20)	0.001^*∗*^	0.126	—
Reading, fine work, and activities of daily living (RFA)				
Mild VF loss	84.31 (16.22)	—	0.026^*∗*^	<0.001^*∗*^
Moderate VF loss	74.78 (19.87)	0.026^*∗*^	—	0.026^*∗*^
Severe VF loss	55.38 (30.33)	<0.001^*∗*^	0.026^*∗*^	—
Total VRQOL				
Mild VF loss	81.55 (13.00)	—	0.014^*∗*^	<0.001^*∗*^
Moderate VF loss	73.08 (15.35)	0.014^*∗*^	—	0.016^*∗*^
Severe VF loss	57.47 (23.22)	<0.001^*∗*^	0.016^*∗*^	—
Worse eye				
General vision and lighting (GL)				
Mild VF loss	75.60 (13.97)	—	0.664	0.020^*∗*^
Moderate VF loss	78.17 (19.91)	0.664	—	0.006^*∗*^
Severe VF loss	63.94 (22.31)	0.020^*∗*^	0.006^*∗*^	—
Mobility (M)				
Mild VF loss	89.32 (11.36)	—	0.366	<0.001^*∗*^
Moderate VF loss	84.72 (15.88)	0.366	—	0.003^*∗*^
Severe VF loss	69.32 (22.16)	<0.001^*∗*^	0.003^*∗*^	—
Psychological adjustment (PA)				
Mild VF loss	81.25 (12.25)	—	0.271	0.005^*∗*^
Moderate VF loss	78.20 (14.25)	0.271	—	0.050
Severe VF loss	67.65 (20.35)	0.005^*∗*^	0.050	—
Reading, fine work, and activities of daily living (RFA)				
Mild VF loss	86.96 (14.56)	—	0.241	0.001^*∗*^
Moderate VF loss	81.00 (17.07)	0.241	—	0.057
Severe VF loss	67.65 (27.31)	0.001^*∗*^	0.057	—
Total VRQOL				
Mild VF loss	83.33 (10.86)	—	0.509	0.001^*∗*^
Moderate VF loss	80.14 (15.10)	0.509	—	0.015^*∗*^
Severe VF loss	66.85 (20.88)	0.001^*∗*^	0.015^*∗*^	—

VF loss was classified according to mean deviation (MD) into mild VF loss: −6 dB < MD < −3 dB, moderate VF loss: −12 dB < MD < −6 dB, and severe VF loss: MD < −12 dB; MD was determined using the Humphrey central 30-2 threshold test (^*∗*^ *P* < 0.05).

**Table 4:** Visual function score of different glaucoma type groups.

	Number of patients	Binocular weighted average BCVA mean (SD)	Binocular MD (dB value) mean (SD)	VRQOL score mean (SD)
Primary angle closure glaucoma	118	0.50 (0.53)	16.58 (12.70)	92.02 (23.83)
Primary open angle glaucoma	84	0.47 (0.52)	20.33 (10.96)	92.17 (24.46)

BCVA: best correct visual acuity; SD: standard deviation; MD: mean deviation; VRQOL: vision health-related quality of life.

**Table 5:** Multiple impact factors that influence VRQOL score of glaucoma patients.

	Mean (SD) or number	*t* or *b*	*P*
Age (year)	69.49 (12.04)	−0.793	0.430
Glaucoma duration (year)	5.72 (6.79)	−1.380	0.171
Education (year)	10.61 (4.28)	0.099	0.921
Binocular MD (dB value)	18.14 (12.09)	−1.878	0.063^*∗∗*^
Binocular weighted average logMAR BCVA	0.48 (0.52)	−10.699	<0.001^*∗*^
Married (yes/no)	152/50	−0.603	0.311
Urban (yes/no)	190/12	1.606	0.232
More than secondary school degree (yes/no)	116/86	0.951	0.052^*∗∗*^
Both eyes MD < −12 dB (yes/no)	58/144	−1.556	0.004^*∗*^
At least 3 years of glaucoma history (yes/no)	102/100	−0.304	0.538
Diabetes mellitus (yes/no)	28/174	−0.714	0.301
Stroke (yes/no)	16/186	−2.240	0.016^*∗*^

VRQOL = vision health-related quality of life; SD = standard deviation; MD = mean deviation; BCVA = best corrected visual acuity (^*∗*^ *P* < 0.05, ^*∗∗*^0.05 < *P* < 0.10).
